# Design, synthesis and antiproliferative activity of oxadiazole derivatives as potent glycogen synthase kinase-3/histone deacetylase 6 dual inhibitors

**DOI:** 10.1080/14756366.2026.2627711

**Published:** 2026-02-23

**Authors:** Changchun Ye, Zilu Chen, Jiantao Jiang, Jianzhong Li, Ranran Kong, Shiyuan Liu, Xin Chen, Zhengshui Xu

**Affiliations:** a Department of Thoracic Surgery, The Second Affiliated Hospital of Xi’an Jiaotong University, Xi’an, Shaanxi, China; b Department of General Surgery, The First Affiliated Hospital of Xi’an Jiaotong University, Xi’an, Shaanxi, China; c Shaanxi Key Labotory of Natural Products and Chemical Biology, College of Chemistry and Pharmacy, Northwest A&F University, Yangling, P.R. China; d Key Laboratory of Surgery Critical Care and Life Support, Ministry of Education, Xi’an Jiaotong University, Xi’an, Shaanxi, China

**Keywords:** Antiproliferative, dual inhibitor, GSK3, HDAC6, oxadiazole

## Abstract

A series of oxadiazole-based dual inhibitors targeting GSK3 and HDAC6 were rationally designed by integrating key pharmacophores into a single molecule. Among these derivatives, 4-(((5-(benzo[*d*][1, 3]dioxol-5-yl)-1,3,4-oxadiazol-2-yl)thio)methyl)-*N*-hydroxybenzamide (**15i**) was identified as the most potent compound with IC_50_ of 5.50, 69 nM and 88 nM against HDAC6, GSK3*α* and GSK3*β*, respectively. **15i** also exhibited potent cytotoxicity against the AGS cancer cell line, with IC_50_ values in the submicromolar range. Molecular docking simulation confirmed that **15i** fitted well into the active sites of both HDAC6 and GSK3*β*. These findings establish compound **15i** as a promising candidate for further evaluation.

## GRAPHICAL ABSTRACT

**Figure:**
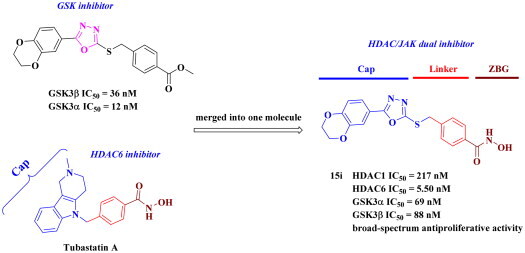

## Introduction

**Figure 1.**
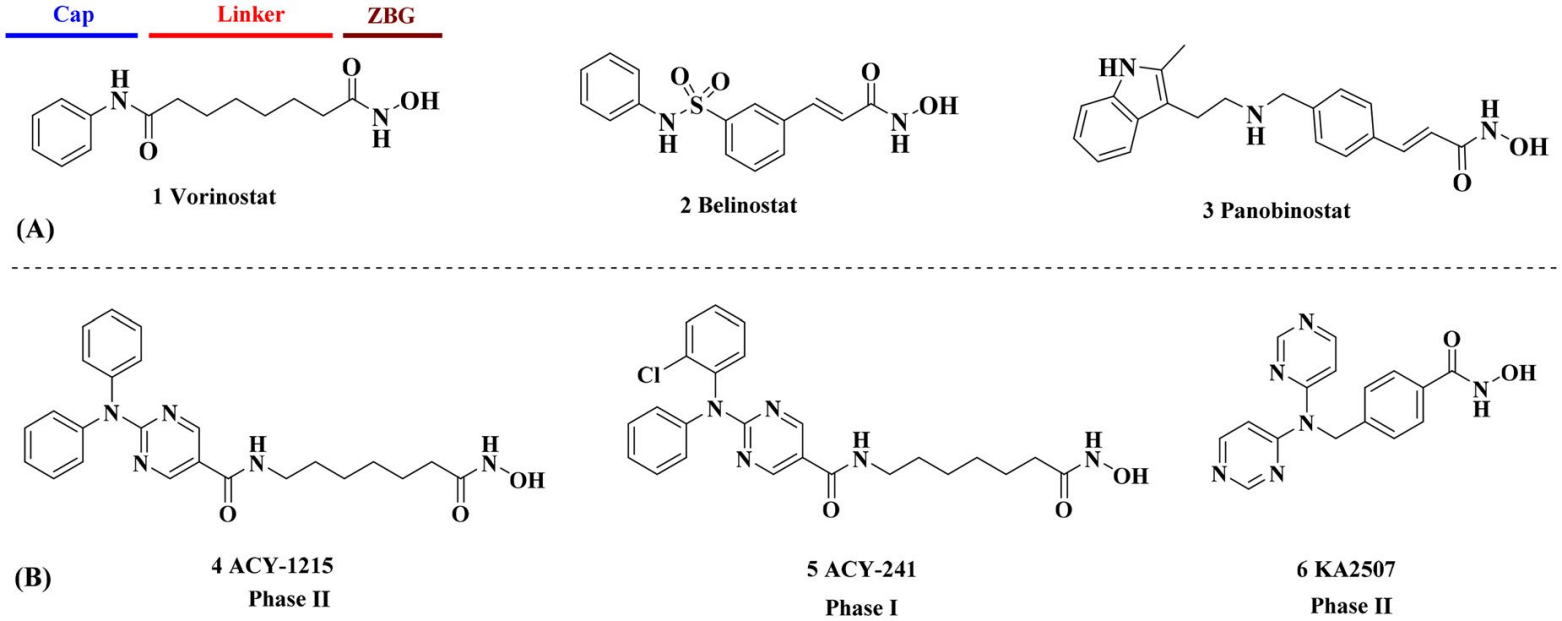
(A) Approved non-selective hydroxamic acid-based HDAC inhibitors; (B) clinical HDAC6 inhibitors.

Histone deacetylases (HDACs), which control the acetylation levels of nuclear proteins and cytoplasmic proteins, have been identified as promising targets for cancer therapy^1–4^. The classical 11 zinc-dependent isoforms comprise class I (HDAC1, HDAC2, HDAC3, HDAC8), class IIa (HDAC4, 5, 7, 9), class IIb (HDAC6, 10), and class IV (HDAC11)^5^^,^^6^. The anticancer mechanism of HDAC inhibition generally included reduced cell motility/migration, invasion, induction of apoptosis, angiogenesis, and blocking of DNA repair. HDAC6, which is primarily localised in the cytoplasm, exhibits distinct characteristics^7^^,^^8^. It features two tandem catalytic domains and acts directly on a host of cytosolic proteins and substrates such as *α*- and *β*-tubulin, heat shock protein, assembled micro-tubules and cortactin^9–11^, thereby regulating processes central to tumorigenesis. Besides, the binding of ubiquitin by unique zinc-finger domain enables HDAC6 regulate protein clearance and degradation. The favourable safety profile and reduced toxicity makes the development of HDAC6 inhibitor (HDAC6i) become a hot research in cancer treatment^7^^,^^12^. The pharmacophore of HDACis is well-established: a capping motif occupying the outside of the protein’s active pocket, a zinc-binding group (ZBG) chelating the catalytically active zinc ion, and a linker chain connecting the above two parts (Figure 1)^13–17^. Such a pharmacophore model usually applies to all isoforms due to the highly conserved nature of HDAC family^18–20^. Hydroxamic acid is the most commonly used ZBG, exemplified by approved *pan*-HDACis such as vorinostat (**1**, SAHA)^21^, belinostat (**2**)^22^ or panobinostat (**3**)^23^, and clinical selective HDAC6i such as ACY-1215 (**4**)^24^, ACY-241 (**5**)^25^ or KA2507 (**6**)^26^.

**Figure 2.**
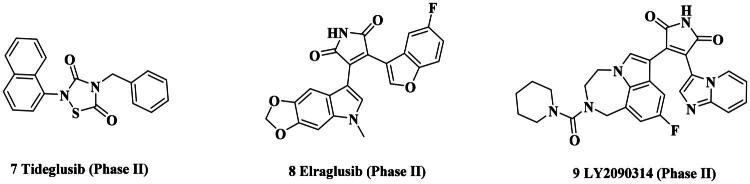
Representative clinical GSK3 inhibitors.

Glycogen synthase kinase-3 (GSK-3) is an evolutionarily conserved serine/threonine kinase with an important role in various cellular processes, including cell proliferation, differentiation, apoptosis, and metabolism^27^^,^^28^. GSK3 has two distinct isoforms in mammals, *α* and *β*, which share high homology within their internal kinase domain^29^. Both GSK-3 isoforms are implicated in various cancer types, including colorectal, breast, prostate, pancreatic, and haematologic malignancies^30^. In particular, GSK3*β* is involved in multiple signal pathway including Wnt/*β*-catenin, PI3K/PTEN/AKT and Notch. It also functions in DNA repair through phosphorylation of DNA repair factors and affecting their binding to chromatin^31^. Thus far, several GSK-3 inhibitors have entered clinical trials such as Tideglusib (**7**)^32^, Elraglusib (**8**)^33^, and LY2090314 (**9**)^34^, as shown in Figure 2. Notably, Elraglusib had demonstrated encouraging efficacy and favourable safety against pancreatic cancer and other solid tumours in Phase II clinical trials.

The connection between HDACs and GSK3*β* have been highlighted^35–38^. Recent studies suggested the importance of co-inhibiting GSK3*β* and HDAC in cancers. Edderkaoui *et al.* demonstrated that a dual GSK3/HDAC inhibitor killed pancreatic cancer cells synergistically and suppressed pancreatic tumour growth and metastasis in mice^39^. Taylan *et al.* also displayed that dual inhibition of GSK3*β* and HDACs exerted significant antitumor effects in an ovarian cancer mouse model^40^. Meanwhile, the structural flexibility of HDAC inhibitors made them feasible for hybridisation with other inhibitors, a design strategy supported by numerous cases^41–47^. Consequently, developing dual GSK3/HDAC inhibitors was a rational and viable drug discovery.

## Compound design

**Figure 3.**
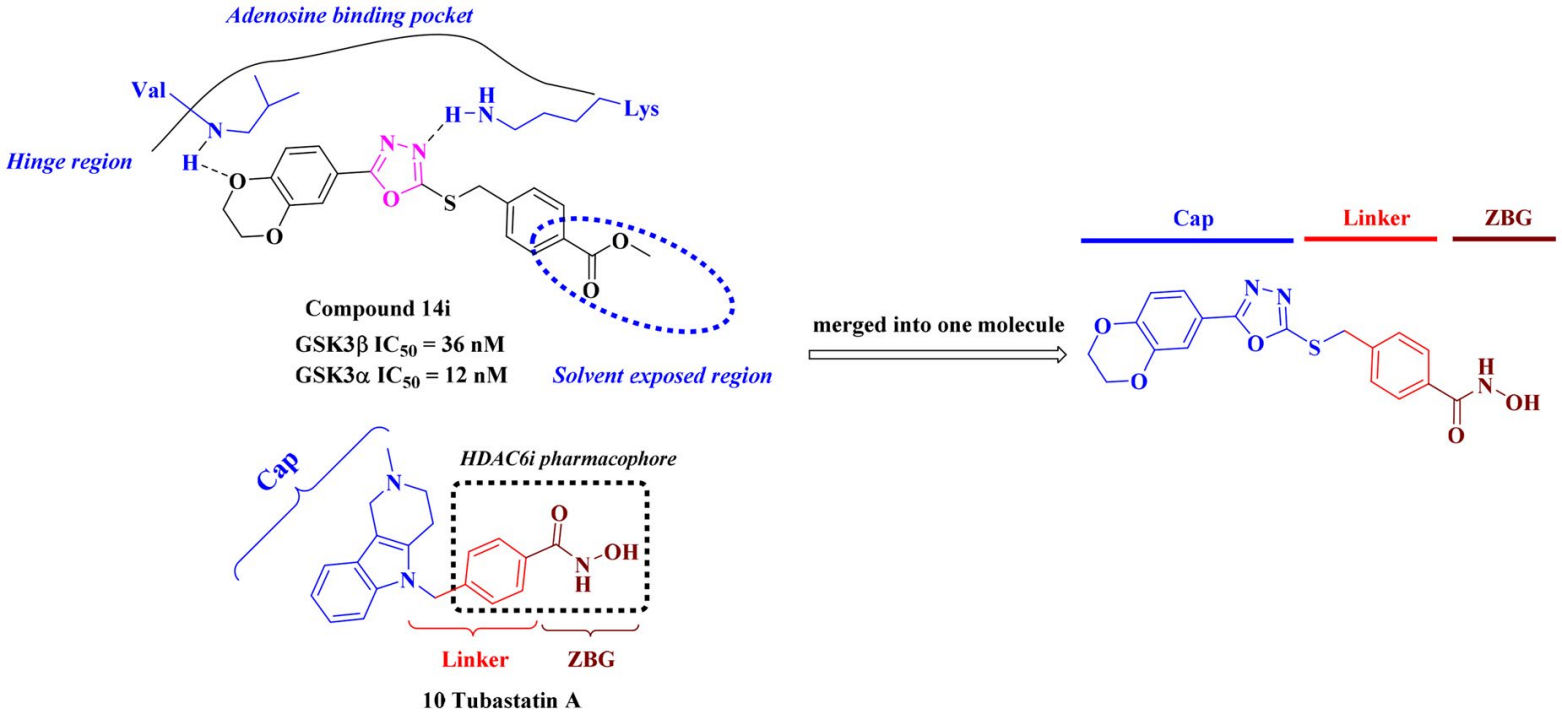
Design of oxadiazole-based GSK3/HDAC6 dual inhibitors.

The common pharmacophore-based design strategy for multi-target drugs typically includes conjugated, fused, and merged pharmacophores^48^. 1,3,4-oxadiazole derivatives were reported for their anti-tumour and antioxidant properties^49–51^. In our design, the merged-pharmacophore design for *de novo* GSK3/HDAC dual-inhibitor was inspired by detailed examination of the binding mode of compound **14i** (a known GSK3 inhibitor^52^) with GSK-3*β* (Figure 3). Its oxadiazole-phenyl scaffold occupied the adenosine-binding pocket, with one nitrogen atom forming a hydrogen bond with the protein and one oxygen atom from the dihydrodioxine ring interacting with hinge region. Both phenyl moieties engaged in hydrophobic interactions with the surrounding residues. The ester group did not provide any further interaction with the enzyme, as it was oriented towards the solvent. On the other hand, phenylhydroxamic acid group was a well-established and widely utilised pharmacophore in HDAC6i design, as shown in Tubastatin A (**10,** a highly selective HDAC6i^53^). Consequently, the direct conversion of the ester group in compound **14i** to a hydroxamate afforded a novel inhibitor with potential dual activity against HDAC6 and GSK3. Here, we reported the synthesis, structure–activity relationship (SAR) study and antiproliferative evaluation of these merged oxadiazole derivatives.

## Chemistry

**Scheme 1.**
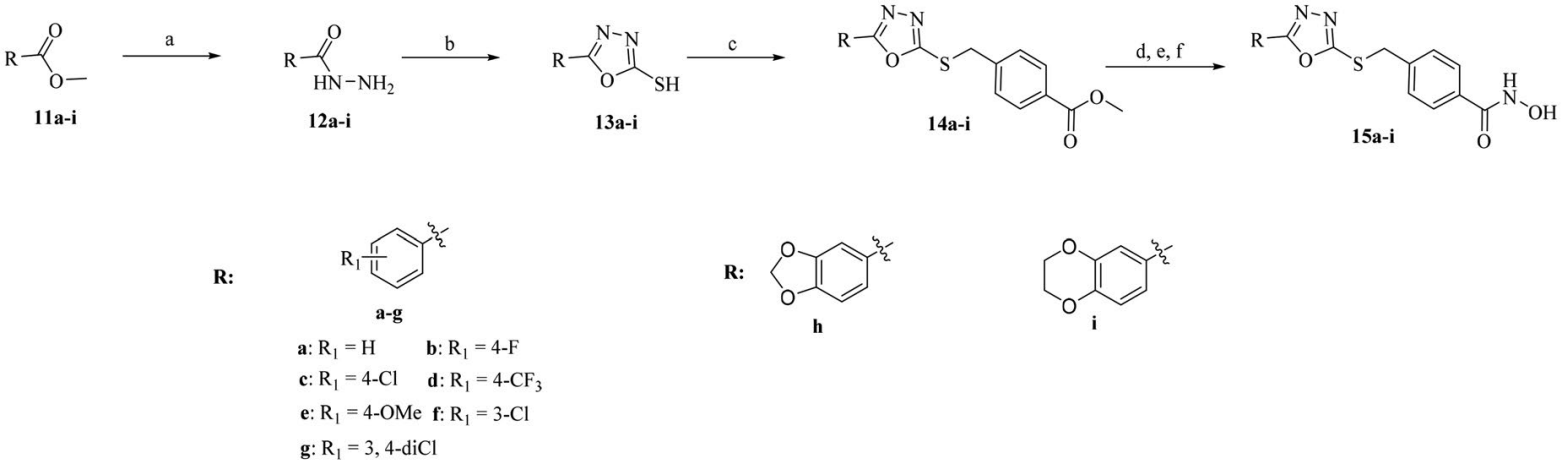
Reagents and conditions: (a) NH_2_NH_2_.H_2_O, EtOH, 78 °C, overnight; (b) CS_2_, Et_3_N, EtOH, reflux, 8–12 h; (c) benzyl halides, 1 N NaOH, anhydrous DMF, r.t., 6–12 h; (d) NaOH, MeOH/H_2_O, r.t., overnight; (e) HATU, DIPEA, DMF, *O*-(tetrahydro-2*H*-pyran-2-yl) hydroxylamine, 0 °C, 6 h; (f) *p*-TsOH.H_2_O, MeOH, r.t., overnight.

The primary modification site for these oxadiazole derivatives was focused on the capping phenyl. Compounds **15a–i** were synthesised according to Scheme 1. Briefly, starting materials **11a–i** were treated with hydrazine hydrate to provide the corresponding hydrazides **12a–i**. Subsequent cyclisation of **12a–i** with carbon disulphide yields the key intermediate **13a–i,** as reported in relevant literatures^52^^,^^54^. Then, **13a–i** reacted with methyl *p*-bromomethylbenzoate under mild conditions via nucleophilic substitution to form intermediates **14a–i**. Direct aminolysis of intermediates **14a–i** was precluded by concomitant oxadiazole ring-opening. Consequently, an alternative one-pot, three-step procedure was adopted. This involved hydrolysis of **14a–i** to the corresponding carboxylic acids, followed by condensation with *O*-(tetrahydro-2*H*-pyran-2-yl)hydroxylamine and subsequent deprotection to furnish the target products **15a–i** in higher overall yield.

## Results and discussion

### HDAC6, GSK3β activities and SAR study of target compounds

GSK3*β* and HDAC6 inhibitory activities of all compounds were evaluated with selective HDAC6is ACY1215 and Tubastatin A, non-selective SAHA, **13i** and Elraglusib as the positives (Table 1). All nine compounds showed higher GSK3*β* activity than Elraglusib with IC_50_ in nanomolar level. **15h** and **15i** demonstrated the most potent inhibition with IC_50_ values of 88 nM and 95 nM, respectively. Replacing the 3, 4-methylenedioxy group with other substituents such as halogens or hydrogen resulted in diminished potency. The conversion of the ester to the hydroxamic acid led to a slightly decline of GSK3*β* activity (**15i** vs **14i**), but all compounds displayed significant potency towards HDAC6. And seven of them exhibited single-digit nanomolar inhibitory activity against HDAC6. **15i** emerged as the most potent compound with IC_50_ of 5.5 nM, comparable to that of ACY1215. For **15e**, **15h** and **15i**, a polar oxygen atom on the capping phenyl enhanced HDAC6 activity compared to halogen substituents or unsubstituted phenyl analog. Based on the above results, it was noteworthy that the substituent effects on the capping phenyl exhibited a favourable alignment between HDAC6 and GSK3*β* activity. And **15i** was identified as the preferred candidate for further investigation.

**Table 1.** Initial evaluation of derivatives against GSK3*β* and HDAC6 (IC_50_a, nM).

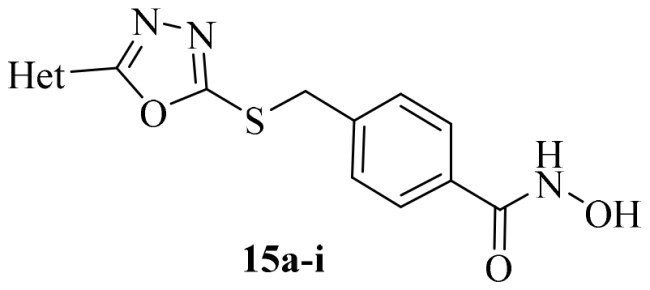			
Compound	Het	HDAC6	GSK3*β*
**15a**	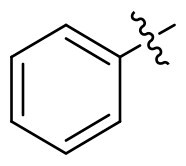	12.3 ± 0.76	430 ± 26
**15b**	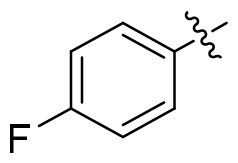	8.60 ± 0.44	340 ± 30
**15c**	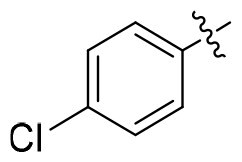	9.00 ± 0.50	380 ± 19
**15d**	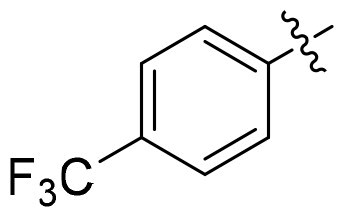	7.10 ± 0.33	225 ± 10
**15e**	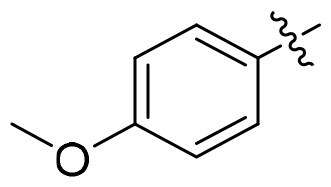	6.00 ± 0.20	176 ± 9.7
**15f**	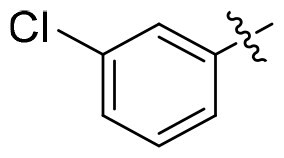	11.0 ± 0.85	268 ± 21
**15g**	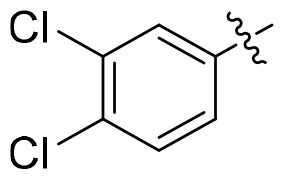	8.45 ± 0.42	130 ± 8.5
**15h**	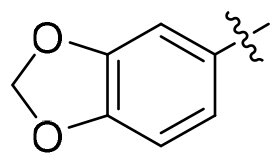	7.06 ± 0.55	95 ± 3.0
**15i**	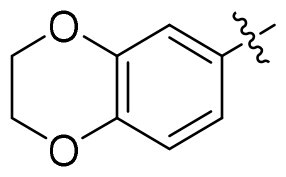	5.50 ± 0.23	88 ± 5.4
**ACY1215**	/	5.13 ± 0.28	/
**Tubastatin A**	/	14.3 ± 0.41	/
**SAHA**	/	7.10 ± 0.15	/
**14i**	/	/	36 ± 1.1
**Elraglusib**	/	/	710 ± 50

^a^IC_50_ values for enzymatic inhibition of HDAC6 and GSK3*β* enzymes. We ran experiments in duplicate, SD <15%. Assays were performed by Reaction Biology Corporation (Malvern, PA, USA).

**15i** was evaluated for isoform selectivity against other HDACs. As depicted in Table 2, **15i** inhibited HDAC1 with IC_50_ of 217 nM, leading to a 40∼fold selectivity index. Besides, **15i** also showed moderate inhibition against HDAC2 and 3, weak activity against HDAC7 and 8, and no activity against HDAC4 and 5. In kinase panel screening, **15i** exhibited comparable inhibitory activity against GSK-3*α* (IC_50_ = 69 nM). It was also tested against five common kinases such as cyclin-dependent kinase 4 (CDK4)/cyclin D1, CDK6/cyclin D1, Fms-like tyrosine kinase 3 (FLT3), breakpoint cluster region-Abelson (BCR-ABL) and epidermal growth factor receptor (EGFR). As shown in Table 3, **15i** showed weak off-target effects against the profiled kinases.

**Table 2.** Complete characterisation of **15i** at other HDAC enzymes (IC_50_a, nM).

Compound	15i	SAHA	Compound	15i	SAHA
HDAC1	217 ± 11	4.37 ± 0.24	HDAC6	5.50 ± 0.23	7.80 ± 0.57
HDAC2	398 ± 14	12.1 ± 1.28	HDAC7	2600 ± 135	>50000
HDAC3	503 ± 25	3.34 ± 0.27	HDAC8	1050 ± 98	1033 ± 62.5
HDAC4	>50000	>50000	HDAC11	2800 ± 161	895 ± 71.6
HDAC5	>50000	>50000			

^a^IC_50_ values for enzymatic inhibition of HDAC enzymes. We ran experiments in duplicate. Assays were performed by Reaction Biology Corporation (Malvern, PA, USA).

**Table 3.** Kinase inhibitory activity of compound **15i**.

Kinase	Inhibition rate%	IC_50_a (nM)
GSK3*β*	80%	88 ± 2.7
GSK3*α*	82%	69 ± 3.0
CDK4/cyclin D1	17%	/
CDK6/cyclin D1	11%	/
FLT3	8%	/
BCR-ABL	10%	/
EGFR	5%	/

^a^IC_50_ values for enzymatic inhibition. We ran experiments in duplicate. Assays were performed by Reaction Biology Corporation (Malvern, PA, USA).

### Molecular simulation

**Figure 4.**
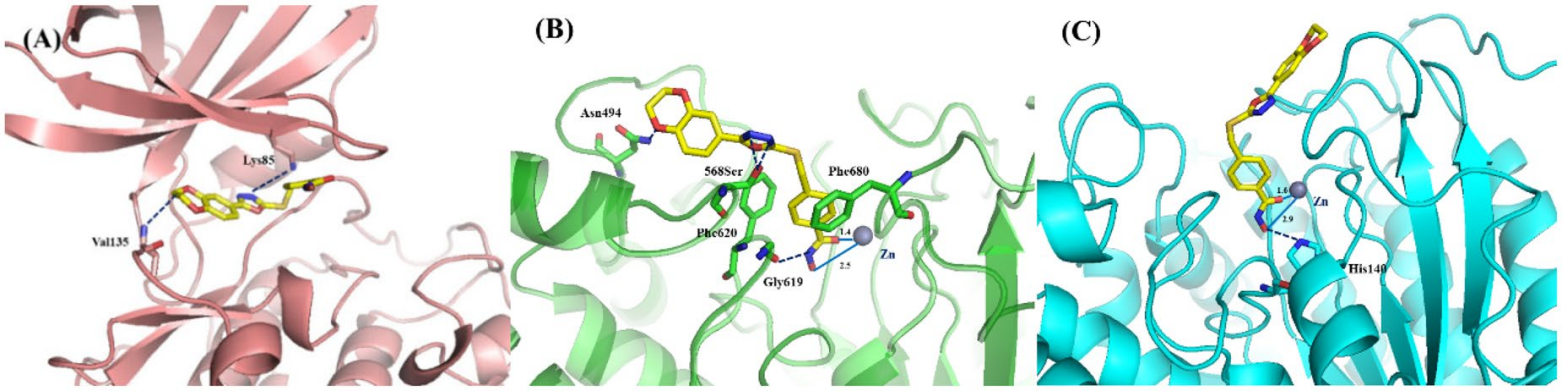
(**A**) Binding model of **15i** (yellow) in the catalytic pocket of GSK-3*β* (PDB code: 3F88). (**B**) Binding model of **15i** (yellow) in the catalytic pocket of human HDAC6 (PDB code: 5EDU). (C) Binding model of **15i** (yellow) in the catalytic pocket of human HDAC1 (PDB code: 5ICN). GSK3*β* protein, HDAC6 protein and HDAC1 was labelled in burgundy, green and light blue, respectively. The hydrogen bonds were labelled in blue. Zinc ion was shown in brown.

Due to the high sequence identity in the active pockets of GSK3*α* and GSK3*β*, GSK3*β* was chosen for molecular docking study because its role in tumorigenesis was more extensively characterised. Compound **15i** was docked into human GSK3*β* and HDAC6 proteins to validate the SAR underlying the enzymatic activity. As outlined in Figure 4A, the phenyl-oxadiazole core of **15i** located in GSK3*β* protein’s adenine-binding site and retained two critical hydrogen bond with the residues of Lys85 and Val135, which was also observed in the binding mode of GSK3*β* with **14i**. The incorporation of hydroxamic acid group did not significantly alter the binding mode of **15i**. The interaction between **15i** and HDAC6 was displayed in Figure 4B, hydroxamate chelated with Zn^2+^ in a bidentate manner with Zn^2+^–O distances of 2.5 Å and 1.4 Å for the OH and C=O groups, respectively. The residue of Gly619 additionally accepted a hydrogen bond from hydroxamate group. The phenyl linker of **15i** embed into the channel between Phe620 and Phe680. Meanwhile, oxadiazole scaffold formed two H-bonds with Ser568 and enabled the capping group to align well with the amino acids on the rim of the binding tunnel. Dihydrodioxine in capping group oriented towards the polar solvent and an oxygen atom interacted with Asn494 by H-bond. Hence, dihydrodioxine group played an important role in binding to both proteins, which rationalises the high enzymatic activity of **15i**. Besides, **15i** was also docked into HDAC1 protein to rationalise the isoform selectivity of these oxadiazole derivatives. As illustrated in Figure 4C, hydroxamic acid group of **15i** also coordinated with zinc ion at the bottom of HDAC1 protein and formed one H-bond with His140. However, the oxadiazole core in cap region failed to form additional H-bonds with HDAC1 in contrast to its binding with HDAC6. Moreover, the distances between hydroxamic acid and zinc ion were greater than those in HDAC6 complex, which indicated a potentially weaker chelation.

### Western blot assay

**Figure 5.**
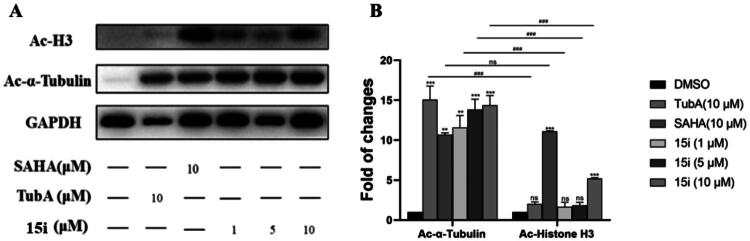
(A). Effect of compound **15i** on the acetylation of histone H3 and *α*-tubulin in AGS cell line (*n* = 3); (B) Densitometry analysis of protein Ac-H3 and Ac-*a*-tubulin. Data are the average of three independent experiments mean ± SD; **p < 0.01, ***p < 0.001 indicated comparison with the control group.

To further confirm the intracellular target specificity of **15i**, gastric cancer cell line AGS were treated with **15i** at concentrations of 1, 5, and 10 μM, along with HDAC6i Tubastatin A (a highly selective HDAC6i) and SAHA at 10 μM as control groups. Selective HDAC6i generally upregulated acetylated *α*-tubulin (Ac-*α*-tubulin) expression with minimal effects on acetylated histone H3 (Ac-H3) level^15^^,^^55^. As shown in Figure 5A and B, **15i** dose-dependently increased acetylated *α*-tubulin level while only inducing moderate Ac-H3 expression. In contrast, the *pan*-inhibitor SAHA significantly elevated both Ac-*α*-tubulin and Ac-H3 levels.

### Antiproliferative activity and mechanism study

**Figure 6.**
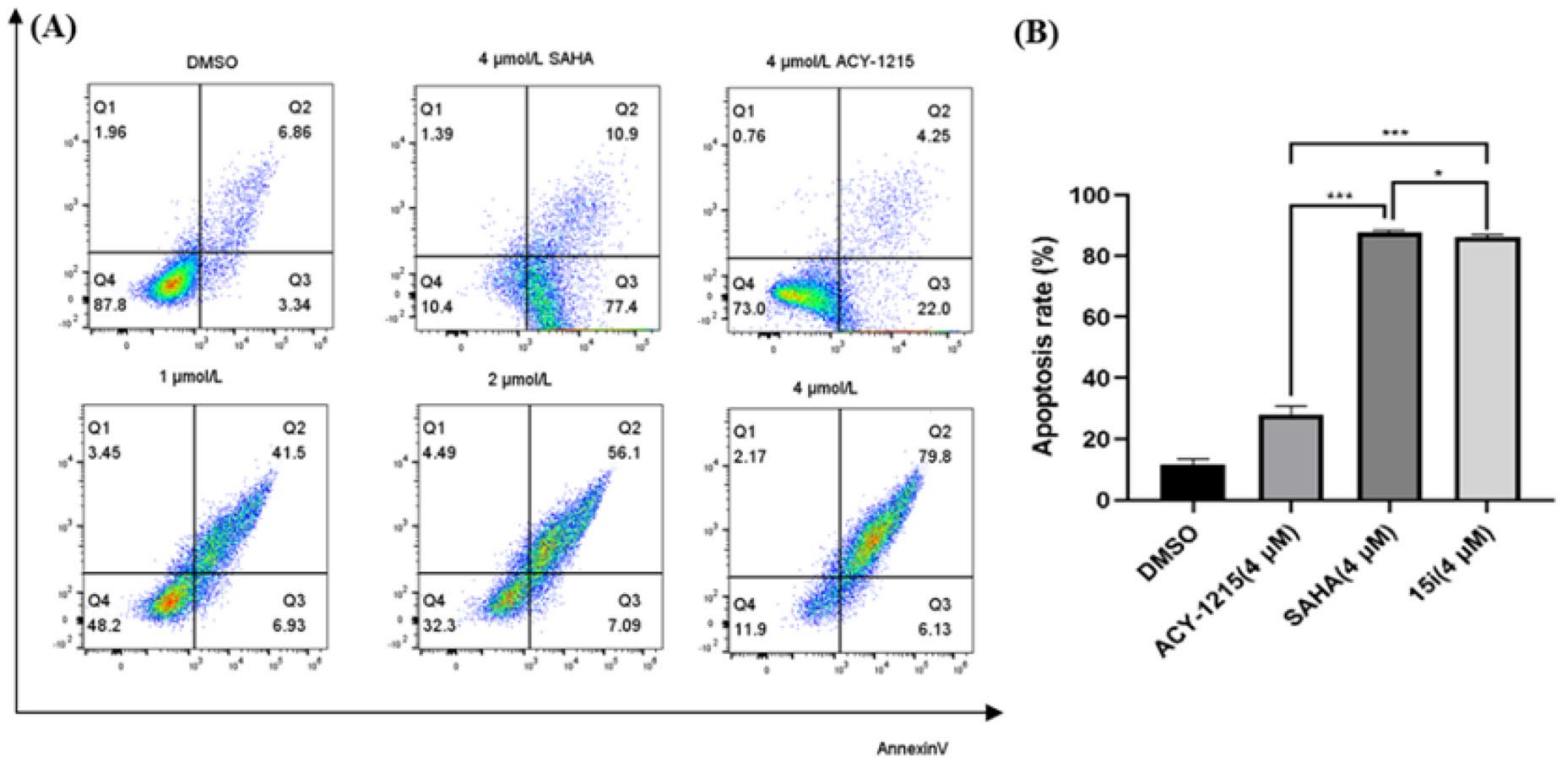
(A) Induction of apoptosis at 48 h by compound **15i** at 1, 2, 4 μM concentration in AGS cell line by flow cytometry analysis with SAHA (4 μM) and ACY1215 (4 μM) as controls. The percentage of cells in each part was indicated. (B) The histogram of SAHA, ACY-1215 and **15i** on AGS cells apoptosis. Data are the mean ± SD of three independent experiments; *p < 0.05, **p < 0.01, ***p < 0.001 indicated comparison with the control group.

**Figure 7.**
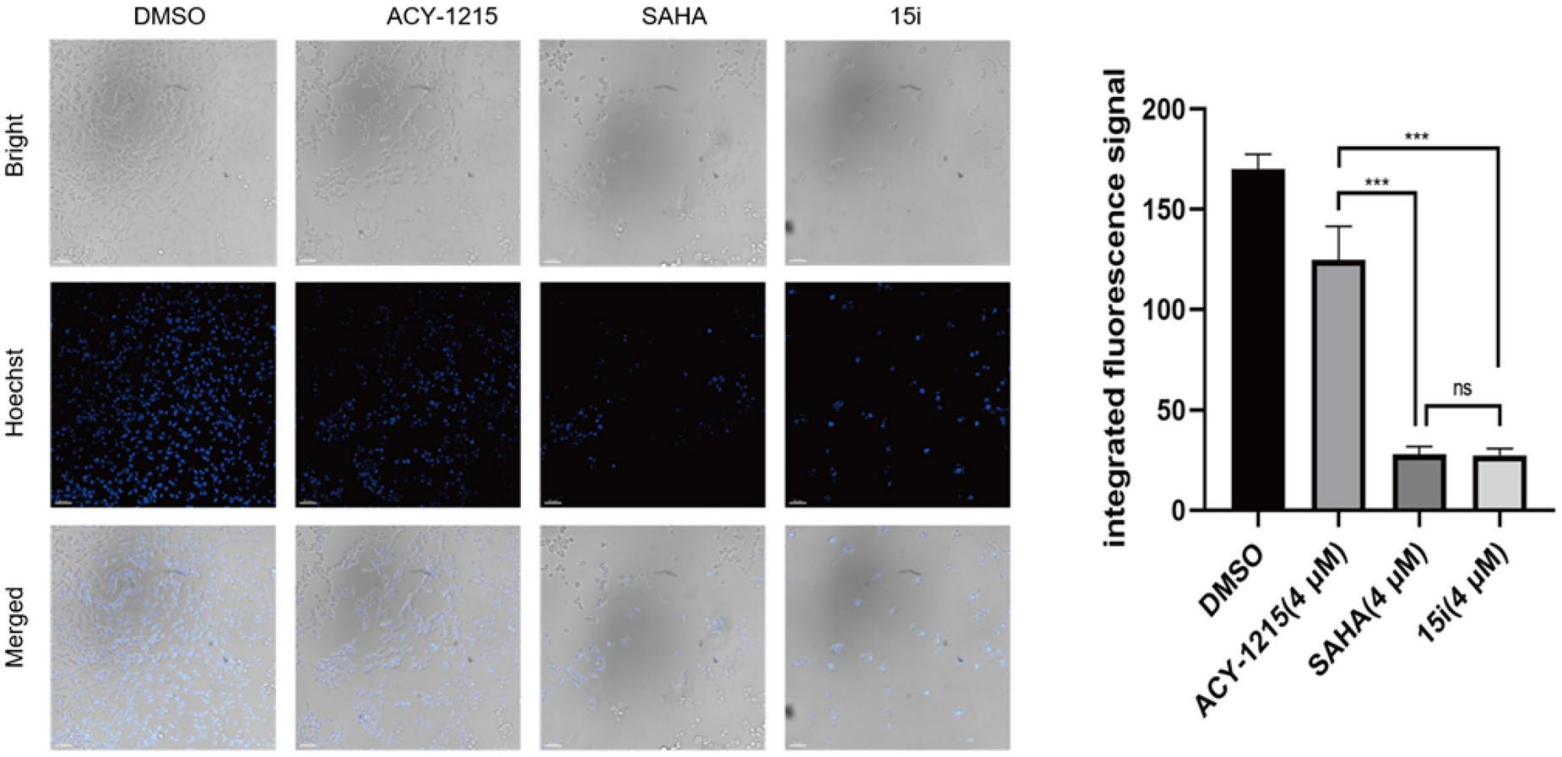
The effect of **15i** on AGS cell apoptosis at 4 μM was determined by Hoechst 33342 staining with SAHA (4 μM) and ACY1215 (4 μM) as controls. Data were subjected to one-way ANOVA. Bars indicate densitometric analysis of immunoblots. Data are the mean ± SD of three independent experiments: *p < 0.05, **p < 0.01, ***p < 0.001 compared with the control group.

**Figure 8.**
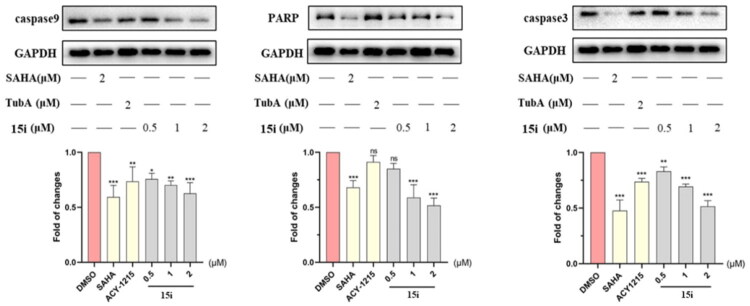
Western blot assay of **15i** on the proteins expression of caspase3, caspase9 and PARP at 0.5, 1, and 2 μM, respectively. The chart showed the densitometric analysis of caspase-3, caspase-9, and PARP expression from Western blots. Data were the average of three independent experiments mean ± SD; *p < 0.05, ** *p* < 0.01, *** *p* < 0.001 indicated comparison with the control group.

Given that gastric cancer is one of the most common and deadly cancers globally^56^, all derivatives were evaluated against gastric cancer cell line AGS in initial *in vitro* screening. As shown in Table 4, all nine compounds demonstrated potent antiproliferative activity with IC_50_ values within single-digit micromolar range, better than ACY-1215 and **14i**. Compared to ACY-1215, *pan*-inhibitor SAHA showed superior antiproliferative activity (IC_50_ = 1.62 μM), which is partially attributed to its non-selective inhibition of Class I HDACs^57^. While dual inhibitors **15b**, **15c**, **15d**, **15f** and **15h** showed comparable activity to SAHA with IC_50_s between 1.2 9 and 1.96 μM. Significantly, **15i** showed the highest potency (IC_50_ = 0.80 μM) yet low activity against HDAC1-3, indicated a synergistic antiproliferative effect from dual GSK3/HDAC6 inhibition. Based on these findings, we subsequently performed a broad-spectrum antitumor screening for compound **15i**. As shown in Table 5, **15i** was evaluated against different cancer cell lines including Hela, RPMI-8226, HepG-2, HT29, MCF-7 and OCI-AML3. All these cells were sensitive to HDAC inhibitors. **15i** demonstrated submicromolar activity against three cell lines RPMI-8226, MCF-7 and OCI-AML3 with IC_50_ values of 0.28, 0.69 and 0.41 μM, respectively. And **15i** also showed single-digit micromolar activity against three others with IC_50_s range from 1.22 to 4.22 μM. The potency was comparable to SAHA and significantly superior to that of ACY-1215. In flow cytometry assay, as expected, **15i** dose-dependently induced AGS cell apoptosis (Figure 6). An apoptosis rate of 85.93% incubated with **15i** at 4 μM was significantly higher than that of ACY1215 (26.25%), comparable to SAHA (88.3%). The pro-apoptotic activity of compound **15i** was further validated by DNA-binding dye Hoechst 33342 assay (Figure 7). Mechanistic study demonstrated that **15i** downregulated the expression of caspase-3, caspase-9, and PARP in a dose-dependent manner, thereby inducing apoptosis (Figure 8).

**Table 4.** Antiproliferative effect of **15a–i** against AGS cell line (IC_50_a, μM).

Compd.	IC_50_^a^	Compd.	IC_50_
**SAHA**	1.62 ± 0.07	**15d**	0.94 ± 0.02
**ACY-1215**	8.02 ± 0.30	**15e**	6.34 ± 0.26
**14i**	9.50 ± 0.55	**15f**	1.92 ± 0.09
**15a**	2.55 ± 0.09	**15g**	3.83 ± 0.16
**15b**	1.96 ± 0.15	**15h**	1.73 ± 0.20
**15c**	1.29 ± 0.11	**15i**	0.80 ± 0.04

*^a^*IC_50_ values are averages of three independent experiments, SD <15%.

**Table 5.** Antiproliferative screen of **15i** against other cell line (IC_50_a, μM).

Tumour type	Cell line	15i	SAHA.	ACY1215
Cervical cancer	Hela	1.22 ± 0.06	1.16 ± 0.05	10.40 ± 0.42
Multiple myeloma	RPMI-8226	0.28 ± 0.01	0.43 ± 0.02	1.50 ± 0.14
Liver cancer	HepG-2	1.82 ± 0.08	4.18 ± 0.29	14.42 ± 1.18
Colon cancer	HT29	2.34 ± 0.08	2.63 ± 0.09	9.85 ± 0.25
Breast cancer	MCF-7	0.69 ± 0.07	0.31 ± 0.02	6.24 ± 0.56
Leukemia	OCI-AML3	0.41 ± 0.01	0.64 ± 0.03	5.24 ± 0.04

^a^IC_50_ values are averages of three independent experiments, SD <15%.

## Conclusion

Given the complexity of tumorigenesis, a series of dual-target inhibitors were designed based on the biological synergy between GSK3 kinase and HDAC6 in our research. Unlike common design for kinase/HDAC dual inhibitors that fused two pharmacophores together via a linker, we utilised an oxadiazole core to elegantly merge key pharmacophores with maximal overlap. The resulting lean molecular structure was a highlight of this study. Enzymatic assays confirmed that all synthesised derivatives exhibited potent HDAC6 inhibition and moderate activity against GSK3. Screening against other relevant targets showed that this class of compounds had minimal off-target activity. Cell-based assays demonstrated that these dual-target inhibitors exhibited significantly better antiproliferative effect against a panel of cancer cell lines than clinical HDAC6i ACY-1215. These results indicated these dual GSK3/HDAC6 inhibitors hold greater potential than single-target inhibitors. Currently, further structural modifications and *in vivo* evaluation are underway.

## Experimental section

### Chemistry

All of the starting reagents were purchased and were used with no additional purification. All of the mentioned yields were for isolated products. Melting points were determined in open capillaries on a WRS-1A digital melting point apparatus (Shenguang).^1^H-NMR spectras were detected on a Bruker DRX–400 (400 MHz) using TMS as internal standard. High resolution mass spectra were obtained from Thermo Scientific Q Exactive. The chemical shifts were reported in ppm (*δ*) and coupling constants (*J*) values were given in Hertz (Hz). The purities of all target compounds were tested by HPLC to be > 95.0%. HPLC analysis was performed at room temperature using an Agilent Eclipse XDB-C18 (250 mm × 4.6 mm) and plotted at 254 nm by 30% MeOH/H_2_O as a mobile phase.

### General procedure for the synthesis of compounds 15a–i

Compounds **14a**–**i** were synthesised according to the standard procedure in literature^52^. Then, **15a**–**i** were synthesised by one-pot, three-step synthesis: (i) To a solution of various esters **14a**–**i** (1 equiv) in methanol and H_2_O (v/v = 1: 1) was added NaOH (3 equiv) The mixture was stirred at room temperature. The reaction was monitored by TLC until the maximum conversion. After neutralised with acetic acid, the formed precipitate was collected by filtration, washed with water, and dried *in vacuo* to give compounds for next step; (ii) At 0 °C, compounds (1 equiv) from step (i) were added to a solution of DIPEA (4 equiv) and HATU (1.1 equiv) in DMF. After 30 min, *O*-(tetrahydro-2*H*-pyran-2-yl)hydroxylamine (1 equiv) was added. The mixture was stirred for additional 4–6 h. After Then, the mixture was poured into water and extracted with EtOAc. The combined organic extracts were washed with brine, and concentrated *in vacuo*; (iii) To a solution of compounds (1 equiv) from step (ii) in methanol was added TsOH^.^H_2_O (0.2 equiv), and the mixture was stirred at room temperature for overnight. After removal of volatiles, residues were purified by chromatography on a silica gel column.

### N-hydroxy-4-(((5-phenyl-1, 3, 4-oxadiazol-2-yl)thio)methyl)benzamide (15a)

Yellow solid, yield 69.6%, m.p.: 180.3–181.9 °C.^1^H NMR (400 MHz, DMSO-*d*_6_) δ 11.19 (s, 1H), 9.03 (s, 1H), 7.95 (dd, *J* = 8.0, 1.5 Hz, 2H), 7.71 (d, *J* = 8.3 Hz, 2H), 7.64 − 7.54 (m, 5H), 4.62 (s, 2H). ^13^C NMR (101 MHz, DMSO-*d*_6_) δ 165.35, 163.89, 163.20, 140.01, 132.20, 132.13, 129.51, 129.10, 127.18, 126.45, 122.99, 35.44. HR-MS (ESI, m/z): Calcd for 328.07504. (C_16_H_14_N_3_O_3_S^+^ [M + H]^+^). Found 328.07462.

### N-hydroxy-4-(((5–(4-(trifluoromethyl)phenyl)-1,3,4-oxadiazol-2-yl)thio)methyl)benzamide (15b)

Yellow solid, yield 67.3%, m.p.: 201.3–203.1 °C. ^1^H NMR (400 MHz, DMSO-*d*_6_) δ 11.19 (s, 1H), 9.03 (s, 1H), 8.17 (d, *J* = 8.2 Hz, 2H), 7.97 (d, *J* = 8.3 Hz, 2H), 7.72 (d, *J* = 8.2 Hz, 2H), 7.57 (d, *J* = 8.2 Hz, 2H), 4.65 (s, 2H). ^13^C NMR (101 MHz, DMSO-*d*_6_) δ 164.29, 164.16, 163.87, 139.87, 132.23, 131.72, 131.40, 129.10, 127.28, 127.18, 126.75, 126.44, 126.40, 125.08, 122.37, 35.42. HR-MS (ESI, m/z): Calcd for 396.06242. (C_17_H_13_F_3_N_3_O_3_S^+^ [M + H]^+^). Found 396.06210. (C_17_H_13_F_3_N_3_O_3_S^+^ [M + Na]^+^). Found 418.04404.

### 4-(((5-(4-chlorophenyl)-1,3,4-oxadiazol-2-yl)thio)methyl)-N-hydroxybenzamide (15c)

Yellow solid, yield 60.3%, m.p.:178.5–180.1 °C. ^1^H NMR (400 MHz, DMSO-*d*_6_) δ 11.19 (s, 1H), 9.03 (s, 1H), 7.96 (d, *J* = 8.6 Hz, 2H), 7.69 (dd, *J* = 17.2, 8.4 Hz, 4H), 7.55 (d, *J* = 8.2 Hz, 2H), 4.62 (s, 2H). ^13^C NMR (101 MHz, DMSO-*d*_6_) δ 164.58, 163.87, 163.47, 139.93, 136.79, 132.20, 129.64, 129.08, 128.24, 127.16, 121.87, 35.42. HR-MS (ESI, m/z): Calcd for 384.01801. (C_16_H_12_NaCl^35^N_3_O_3_S^+^ [M + Na]^+^). Found 384.01779.

### N-hydroxy-4-(((5–(4-methoxyphenyl)-1,3,4-oxadiazol-2-yl)thio)methyl)benzamide (15d)

White solid, yield 65.6%, m.p.: 153.2–154.9 °C. ^1^H NMR (400 MHz, DMSO-*d*_6_) δ 11.18 (s, 1H), 9.03 (s, 1H), 7.89 (d, *J* = 8.9 Hz, 2H), 7.70 (d, *J* = 8.3 Hz, 2H), 7.54 (d, *J* = 8.3 Hz, 2H), 7.13 (d, *J* = 8.9 Hz, 2H), 4.60 (s, 2H), 3.85 (s, 3H). ^13^C NMR (101 MHz, DMSO-*d*_6_) δ 165.29, 164.01, 163.88, 162.32, 162.11, 158.62, 142.64, 140.05, 132.16, 129.05, 128.31, 127.15, 115.31, 114.93, 55.57, 35.46. HR-MS (ESI, m/z): Calcd for 380.06755. (C_17_H_15_NaN_3_O_4_S^+^ [M + Na]^+^). Found 380.06705.

### 4-(((5–(4-fluorophenyl)-1,3,4-oxadiazol-2-yl)thio)methyl)-N-hydroxybenzamide (15e)

White solid, yield 63.5%, m.p.: 189.7–191.4 °C. ^1^H NMR (400 MHz, DMSO-*d*_6_) δ 11.19 (s, 1H), 9.04 (s, 1H), 8.03 (dd, *J* = 8.9, 5.3 Hz, 2H), 7.72 (d, *J* = 8.2 Hz, 2H), 7.56 (d, *J* = 8.2 Hz, 2H), 7.45 (t, *J* = 8.9 Hz, 2H), 4.63 (s, 2H). ^13^C NMR (101 MHz, DMSO-*d*_6_) δ 165.39, 164.60, 163.88, 163.20, 139.96, 132.19, 129.23, 129.14, 129.07, 127.16, 116.85, 116.63, 35.43. HR-MS (ESI, m/z): Calcd for 368.04756. (C_16_H_12_NaFN_3_O_3_S^+^ [M + Na]^+^). Found 368.04724.

### 4-(((5–(3, 4-dichlorophenyl)-1,3,4-oxadiazol-2-yl)thio)methyl)-N-hydroxybenzamide (15f)

Yellow solid, yield 64.8%, m.p.: 183.6–185.1 °C. ^1^H NMR (400 MHz, DMSO-*d*_6_) δ 11.19 (s, 1H), 9.04 (s, 1H), 8.15 (d, *J* = 1.9 Hz, 1H), 7.93 (dd, *J* = 8.4, 2.0 Hz, 1H), 7.87 (d, *J* = 8.4 Hz, 1H), 7.71 (d, *J* = 8.2 Hz, 2H), 7.56 (d, *J* = 8.2 Hz, 2H), 4.64 (s, 2H). ^13^C NMR (101 MHz, DMSO-*d*_6_) δ 163.98, 163.65, 139.93, 134.78, 132.38, 132.21, 131.87, 129.12, 128.12, 127.17, 126.54, 123.48, 35.38. HR-MS (ESI, m/z): Calcd for 417.9790. (C_16_H_11_NaCl_2_N_3_O_3_S^+^ [M + Na]^+^). Found 417.9787.

### 4-(((5–(3-chlorophenyl)-1,3,4-oxadiazol-2-yl)thio)methyl)-N-hydroxybenzamide (15 g)

Yellow solid, yield 66.9%, m.p.: 182.1–183.8 °C. ^1^H NMR (400 MHz, DMSO-*d*_6_) δ 11.20 (s, 1H), 9.05 (s, 1H), 7.97 − 7.91 (m, 2H), 7.71 (d, *J* = 8.2 Hz, 3H), 7.63 (t, *J* = 7.9 Hz, 1H), 7.56 (d, *J* = 8.2 Hz, 2H), 4.63 (s, 2H).^13^C NMR (101 MHz, DMSO-*d*_6_) δ 164.23, 163.78, 139.97, 134.12, 132.21, 131.92, 131.55, 129.12, 127.18, 125.99, 125.15, 124.93, 35.39. HR-MS (ESI, m/z): Calcd for 384.01801. (C_16_H_12_NaCl^35^N_3_O_3_S^+^ [M + Na]^+^). Found 384.01755.

### 4-(((5–(2, 3-dihydrobenzo[b][1,4]dioxin-6-yl)-1,3,4-oxadiazol-2-yl)thio)methyl)-N-hydroxybenzamide (15h)

White solid, yield 58.6%, m.p.: 176.2–177.7 °C. ^1^H NMR (400 MHz, DMSO-*d*_6_) δ 11.20 (s, 1H), 9.05 (s, 1H), 7.70 (d, *J* = 8.2 Hz, 2H), 7.54 (d, *J* = 8.2 Hz, 2H), 7.44 − 7.39 (m, 2H), 7.05 (d, *J* = 8.4 Hz, 1H), 4.59 (s, 2H), 4.32 (d, *J* = 4.0 Hz, 4H). ^13^C NMR (101 MHz, DMSO-*d*_6_) δ 165.00, 163.86, 162.49, 146.76, 143.86, 140.03, 132.15, 129.03, 127.13, 119.99, 118.20, 115.91, 115.09, 64.46, 64.09, 35.41. HR-MS (ESI, m/z): Calcd for 394.04681. (C_18_H_16_N_3_O_5_S^+^ [M + Na]^+^). Found 394.04603.

### 4-(((5-(Benzo[d][1, 3]dioxol-5-yl)-1,3,4-oxadiazol-2-yl)thio)methyl)-N-hydroxybenzamide (15i)

Yellow solid, yield 62.9%, m.p.: 186.5–188.2 °C. ^1^H NMR (400 MHz, DMSO-*d*_6_) δ 11.18 (s, 1H), 9.02 (s, 1H), 7.70 (d, *J* = 8.2 Hz, 2H), 7.56 − 7.48 (m, 3H), 7.44 (d, *J* = 1.6 Hz, 1H), 7.11 (d, *J* = 8.2 Hz, 1H), 6.16 (s, 2H), 4.60 (s, 2H). ^13^C NMR (101 MHz, DMSO-*d*_6_) δ 165.15, 163.87, 162.51, 150.51, 148.16, 140.04, 132.17, 129.06, 127.15, 121.77, 116.63, 109.20, 106.18, 102.18, 35.41. HR-MS (ESI, m/z): Calcd for 408.06246. (C_17_H_14_N_3_O_5_S^+^ [M + Na]^+^). Found 408.06201.

### In vitro HDAC enzyme assay

IC_50_ testing of compounds were performed by Reaction Biology Corporation. Detailed methods can be found in our previously published literature^43^.

### Kinase inhibition assay

IC_50_ testing of compounds was performed by Reaction Biology Corporation. Detailed methods can be found in https://www.reactionbiology.com/services/biochemical-assays/kinase-screening/.

### Cell culture and antiproliferative assay

All cells (Procell Life Science and Technology Co., Ltd) were maintained at 37 °C in a humidified atmosphere of 5% CO_2_ in air. The cells were cultured in IMDM medium with 20% FBS, 100 U/mL penicillin and 100 µg/mL streptomycin. Briefly, 100 µL cell suspension or completed medium were plated into 96-well plate (5 × 10^4^ cells per well) for 24 h. The compounds were serially diluted to concentrations of 20, 10, 5, 2.5, 1.25, 0.625 0.313 μM and incubated for 48 h; Then, Alamar blue solution (10%v/v) were pipetted into each well of 96-well plate; and the plate was incubated for an additional 5–6 h. The absorbance (OD) was read at 530/590 nm. Data were normalised to vehicle groups (DMSO) and represented as the means of three independent measurements with standard errors of <20%. The IC_50_ values were calculated using Prism 5.0.

### Apoptosis assay

For flow cytometry assay, detailed methods can be found in our previously published literature^43^.

For Hoechst 33342 staining assay, 1.5 ml AGS cells (3.0 × 10^5^ cells/mL) were seeded and incubated for 24 h, Then, the control and experimental groups were supplemented with 1.5 ml of fresh medium containing 0.1% DMSO and compounds at 4 μM, respectively. After incubated for 48 h, 750 μL Hoechst 33324 (2 μg/mL) was added to each dish and incubated for 20 min. Then, the samples were washed with PBS and examined for specific staining using a laser scanning confocal microscope at 405 nm.

### Western blotting assay

AGS (purchased from Procell Life Science and Technology Co., Ltd; NO. PC-H2023011119; 3 × 10^5^) were seeded overnight and incubated with compound **15i** for 48 h on indicated concentrations. Cell extract was prepared by lysing cultured cells with a mammalian protein extraction reagent supplemented with EDTA-free protease inhibitor for 15 min. Supernatants were collected following centrifugation of lysed cells at 15000*g* for 10 min at 4 °C. Protein samples (15 μg per lane) were resolved by SDS-PAGE and subsequently transferred to PVDF membranes (0.45 μM). Membranes were blocked with 5% non-fat milk in TBST and subsequently incubated overnight at 4 °C with primary antibodies against Ac-H3 (abcom, AB32129) and Ac-*ɑ*-tubulin (Cell Signalling, 2144), PARP, Caspase-9, Caspase-3, and GAPDH. After incubation with a rabbit-derived secondary antibodies, immunoreactive bands were visualised by chemiluminescence. Quantification was performed using ImageJ (v2.0), and data were analysed with GraphPad Prism (v8.0).

### Computational methods

Molecular docking was performed using Sybyl-X 2.0 software (222 S Central Ave Ste 1008, Saint Louis, MO 63105, USA) based on the cocrystal of HDAC6 (PDB: 5EDU). The cavity occupied by trichostatin A was selected as the ligand binding site. For HDAC1, the cocrystal of PDB: 5ICN was used for docking. GLY-ALA-6A0-ARG-HIS was selected as the ligand binding site. For GSK3*β*, the cocrystal of PDB: 3F88 was used for docking. And 5–(1-(4-methoxyphenyl)-1*H*-benzimidazol-6-yl)-1, 3, 4-oxadiazole-2(3*H*)-thione was selected as the ligand binding site. Water molecules outside the binding pocket were excluded. The other docking parameters were kept as default.

## Data Availability

The datasets presented in the current study are available from the corresponding author upon reasonable request.
